# Effect of 26 Years of Intensively Managed *Carya cathayensis* Stands on Soil Organic Carbon and Fertility

**DOI:** 10.1155/2014/857641

**Published:** 2014-01-15

**Authors:** Jiasen Wu, Jianqin Huang, Dan Liu, Jianwu Li, Jinchi Zhang, Hailong Wang

**Affiliations:** ^1^College of Forest Resources and Environment, Nanjing Forestry University, Nanjing 210037, China; ^2^Zhejiang Provincial Key Laboratory of Carbon Cycling in Forest Ecosystems and Carbon Sequestration, Zhejiang Agricultural and Forestry University, Lin'an, Zhejiang 311300, China

## Abstract

Chinese hickory (*Carya cathayensis*), a popular nut food tree species, is mainly distributed in southeastern China. A field study was carried out to investigate the effect of long-term intensive management on fertility of soils under a *C*. *cathayensis* forest. Results showed that after 26 years' intensive management, the soil organic carbon (SOC) content of the A and B horizons reduced by 19% and 14%, respectively. The reduced components of SOC are mainly the alkyl C and O-alkyl C, whereas the aromatic C and carbonyl C remain unchanged. The reduction of active organic matter could result in degradation of soil fertility. The pH value of soil in the A horizon had dropped by 0.7 units on average. The concentrations of the major nutrients also showed a decreasing trend. On average the concentrations of total nitrogen (N), phosphorus (P), and potassium (K) of tested soils dropped by 21.8%, 7.6%, and 13.6%, respectively, in the A horizon. To sustain the soil fertility and *C*. *cathayensis* production, it is recommended that more organic fertilizers (manures) should be used together with chemical fertilizers. Lime should also be applied to reduce soil acidity.

## 1. Introduction

Soil is an essential natural resource for human being, and management practices can significantly influence the land productivity and change soil properties. For example, intensive management of *Phyllostachys praecox* bamboo forests has shown to result in accumulation of nitrogen (N) and phosphorus (P) and soil organic carbon (SOC), enhancement of soil respiration, reduction of soil pH [1, 2], and increased soil respiration [3].

Chinese hickory (*Carya cathayensis* Sarg.) is a plant for production of edible nuts and oil in southeastern China. Planting area is around 83,000 hm^2^ and total yield reached 27,600 t in 2009 [4], mainly in the Tianmushan area at the junction of Zhejiang and Anhui provinces of China (29-30°N, 118–120°E). It is one of the two popular nut food tree species belonging to *Carya* genus. The other is American pecan (*Carya illinoinensis*) [5]. The kernel of the Chinese hickory nuts is a reputed healthy food in China.

*C. cathayensis* cultivation and utilization has a history of over 500 years. Before the 1980s, *C. cathayensis* forests were owned by the village collectives and the trees were naturally grown in wild or semiwild conditions with minimal management. Nut yields were low and from year to year partly due to competition of other tree species and understory weeds. Since 1982, *C. cathayensis* forests were distributed to farmers who gradually adapted the intensive management practices to achieve a high yield and better income. Typically, a compound fertilizer (N : P_2_O_5_ : K_2_O = 15 : 15 : 15) at a rate of 225 kg ha^−1^ was applied twice a year, in mid-March and early September. Since 1990, herbicides such as glyphosate (with an application rate of 22.5 kg ha) were sprayed to control shrubs and understory weeds, which make fruit picking more convenient. Meanwhile, pest control using pesticides has also been implemented. With the development of intensive management practices, the average fruit yield of *C. cathayensis *has risen from 395 kg ha in the early 1980s to 1000 kg hm^−2^ in recent years [6].

Continued development and adaption of advanced management practices, such as grafting, high rate fertilizer use, weeding, and other effective techniques, have greatly helped improve *C. cathayensis* production and economic return to growers [7–9]. However, this intensive management for highly economic profit of nut production can lead to soil degradation. The influence of such intensive management measures on soil fertility and other properties under *C. cathayensis *forest has not been reported in the literature. Therefore, the objectives of this study were to investigate the effects of long-term intensive management on soil organic carbon (SOC) and fertility under *C. cathayensis *stands. This would provide useful information for sustainable management of *C. cathayensis* forests.

## 2. Materials and Methods

The study site was located in Lin'an, northwest of Zhejiang Province, China. It is the major production region of *C. cathayensis*. This area belongs to subtropical monsoon climate with the annual average temperature of 16°C, with the highest of 41.7°C in July and the lowest of −13.3°C in January. The annual average effective accumulated temperature of 5774°C. The annual average precipitation is 1420 mm. The annual average sunshine hour is about 1,774 h, with 235 frost-free days each year. *C. cathayensis* typically grows in hilly areas with altitudes in the range of 150 to 1000 m above sea level. The soils in the area are developed from limestone.

The soil samples were taken in two different years at A (0–26 cm) and B (26–51 cm) horizons from the same permanent plots (Table 1). The first sampling time was during the second national soil survey in China in August 1982. 36 soil samples from the three permanent sampling plots of *C. cathayensis* forest were air-dried, sieved, and stored in the sealed bottle of soil samples in the Soil and Fertilizer Extension Station in Lin'an. The second sampling time from the same plots was in August 2008. Soil samples were collected and processed with the same manner as in August 1982.

All soil samples were analyzed according to soil analysis methods recommended by Lu [10]. Soil pH was measured using a pH meter at a soil : water ratio of 1 : 5. Organic carbon was determined using the sulfuric acid-potassium external heating method. Soil total nitrogen (N) was measured using the Kjeldahl procedure. Acid digestion was used to extract other elements. Total phosphorus (P) was determined with Mo-Sb colorimetry, and total potassium (K) was determined with a flame photometer.

Soil samples from A horizon were further analyzed using cross-polarization magic-angle-spinning (CPMAS) solid-state NMR spectroscopy. Prior to the ^13^C NMR analysis, soil samples were pretreated with HF to remove Fe^3+^ and Mn^2+^ from the soil in order to enhance the signal-to-noise ratio of the instrument. The HF pretreatment followed Mathers et al. [12], 5 g of air-dried soil were weighed and put into a 100 mL plastic centrifuge tube, and 50 mL HF solution (10% v/v) was added to the tube, which were vibrated for 1 h; then the mixture was centrifuged for 10 min (3000 r min^−1^), and the supernatant liquid was removed, and the residue was treated with HF solution continuously. These steps are repeated 8 times, but vibrating time is different (4 × 1 h, 3 × 12 h,1 × 24 h). Soil samples are washed 4 times with double distilled water to remove residue after being treated by HF solution in order to remove HF remaineding in soil samples. The major steps are as follows: firstly, 50 mL double distilled water was added to the tube, and then supernatant liquid was removed after vibrating the tube for 10 min and centrifuging the mixture for 10 min (3000 r min^−1^). These steps are repeated 4 times. Residue treated by HF is dried in an oven at 40°C, and then it is ground to pass through a 60 mesh screen and put in a bag for NMR measurement.

The HF-treated soil samples were subjected to ^13^C NMR analysis with a Bruker (Spectrospin, Rheinstetten, Germany) Avance 300 MHz NMR spectrometer. The experiments were carried out using a 7 mm CPMAS probe, at a carbon frequency of 75 MHz, MAS spinning frequency at 5 kHz, contact time at 2 ms, and recycle delay time at 2.5 s. The external standard used for chemical shift determination was hexamethylbenzene (methyl at 17.33 ppm). According to the literature [2], the NMR spectra were divided into the following four regions representing the different chemical environments of a ^13^C nucleus: alkyl C (0–50 ppm), O-alkyl C (50–110 ppm), aromatic C (110–160 ppm), and carbonyl C (160–220 ppm). Through measuring the area under the curve in each region, we obtained the relative content of the different C fractions. Two indices of organic matter stability were calculated: (1) Alkyl C to O-alkyl C ratio (A/O-A) = C_0–50 ppm_ /C_50–110 ppm_ and (2) aromaticity: C_110–160 ppm_/C_0–220 ppm._ The absolute concentration of the different C fractions was a product of the total soil organic C concentration and relative content of the C fractions [2, 13].

The data presented in this paper are the average of three replicates. A one-way analysis of variation was carried out on the data obtained from the present study, and means were compared using Duncan's multiple range test. The statistical analyses were performed using SPSS 11.5 for Windows (SPSS2003).

## 3. Results

### 3.1. Changes in Soil pH, Soil Organic Carbon, Nitrogen, Phosphorus, and Potassium

Soil analysis results showed that concentrations of the total SOC, N and K, and pH values in A horizon between the two sampling years were significantly (*P* < 0.05) different (Table 2). Total P concentration in A horizon and all parameters in B horizon measured in the 2008 soil samples were substantially lower than those in the 1982 samples, although the differences were not significant statistically (Table 2). After 26 years intensive management, soil pH values decreased by 0.7 units and 0.5 units in A and B horizons, respectively. The SOC content of A and B horizons dropped by 19.0% and 14.1%, respectively, from year 1982 to 2008.

In addition, changes in the fertility indicators of soils under *C. cathayensis* forest stands in the A horizon were more profound than those in B horizon. After 26 years intensive management, the pH values of A horizon and B decrease by 0.7 units and 0.5 units, respectively. This shows a more substantial decrease of pH in A horizon than that in B horizon. Similarly, the total N, P, and K concentrations decreased by 0.55, 0.05, and 0.83 g kg^−1^ in A horizon, respectively, whereas they decreased by 0.30, 0.04, and 0.81 g kg^−1^ in B horizon, respectively (Table 2).

According to a local soil survey report titled “Lin'an Soil” an internal publication of Lin'an Agricultural Bureau in 1984, SOC concentration of the soils in the study area ranged from 22 to 50 g kg^−1^, and soil pH values were >6.5 in the early 1980s. In a more recent survey conducted in 2008, it was found that 77.8% of the soils had less than 20 g kg^−1^ of SOC and 55.6% of the soils had pH < 6.5, and some of the soils had extremely severe conditions (SOC < 12.5 g/g, pH < 6.0) (Table 3).

### 3.2. ^**13**^C CPMAS NMR Spectra of the Soil Organic Carbon

**Figure 1:**
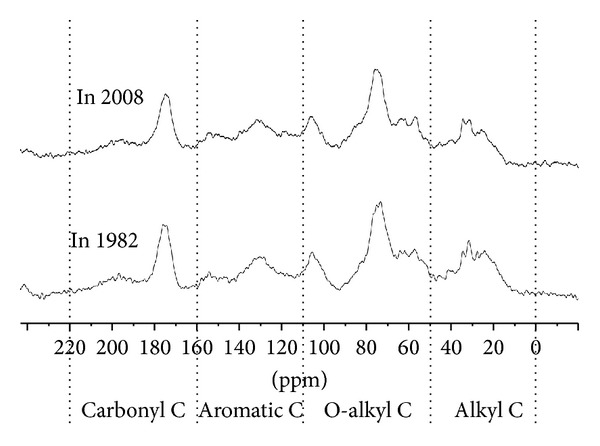
NMR spectra of soil total organic carbon under* Carya cathayensis* Sarg. forest land in different times.

Solid-state ^13^C NMR spectrogram analysis results for SOC in A horizon under *C. cathayensis* forest stand of two different sampling years in plot XQ are shown in Figure 1. The NMR spectrogram includes 4 obvious resonance regions, that is, alkyl C region (0–50 ppm), O-alkyl C region (50–110 ppm), aromatic C region (110–160 ppm), and carbonyl C region (160–220 ppm). There were no significant differences in the SOC forms and the changes in the signal intensity between the two sampling years. The dominant organic C in both sampling years was O-alkyl C which accounted for nearly 50% of the SOC. Carbonyl C contributed to the lowest proportion of the organic C in the SOC. Significant differences in the signal intensity of different carbon forms were observed between the two sampling years. Alkyl C content of the SOC sampled in 1982 was significantly (*P* < 0.05) higher than that sampled in 2008, whereas carbonyl C content of the SOC sampled in 1982 was significantly (*P* < 0.05) lower than that sampled in 2008. Aromatic C content of the SOC sampled in 1982 was higher than that sampled in 2008, and the alkyl C to O-alkyl C ratio (A/O-A) of the SOC sampled in 1982 was lower than that sampled in 2008. However, these differences were not significant statistically. The aromaticity of the SOC sampled in 1982 was significantly (*P* < 0.05) lower than that sampled in 2008 (Table 4).

## 4. Discussion

### 4.1. Effect of Long-Term Intensive Management on Concentrations and ^13^C NMR Spectra of Soil Organic Carbon

Typically, SOC dynamics in forests are mainly influenced by inputs from litter, understory biomass, plant roots, and root exudates and outputs through mineralization [11, 14]. Management practices could have profound effect on SOC buildup. The long-term intensive management of the *C. cathayensis* stands has resulted in reduction of SOC on the study site, such as application of chemical fertilizers and removal of understory vegetation.

Soil organic carbon is one of the most important indices of soil fertility. The changes in SOC contents and compositions reflect the soil quality evolution. Ratio of different components in total SOC may be associated with soil type, climate characteristics, plant species, and management mode [15, 16]. The calculated alkyl C content in A horizon decreased significantly, while the aromatic C contents remained unchanged and the carbonyl C contents have increased slightly. The alkyl C ratio of SOC in A horizon was decreased, but the O-alkyl C, aromatic C, and carbonyl C ratios all increased. As intensive management time is prolonged, A/A-O value becomes lower, which indicated that reduced organic carbon in soil is mainly alkyl C. Thus, reduced components of SOC are mainly the alkyl C and O-alkyl C and the reduction of active organic matter will result in degradation of soil fertility quality [17]. In the process of *C. cathayensis* intensive management, the removal of understory shrubs and weeds leads to the reduction of the litters returning to forest soil, which results in the decrease of SOC. Meanwhile, the bare soil surface under *C. cathayensis* stands has brought about serious soil erosion [18], can accelerate mineralization of alkyl C component in soil, and cause SOC decline.

### 4.2. Soil Degradation

Soil acidification is one of the key factors which impair the soil conditions and cause soil degradation, which would restrain the development of agriculture and forestry in southern China, and human management activities further speed up the acidification process [19]. Long-term application of acidic and physiological acid fertilizer increases the acidity of soil by different degrees. After 8 years of intensive management, the pH value of soil in A horizon under Lei bamboo forest stand decreases by 0.95 units [20]. Ji [21] also showed that the soil pH decreased from 5.6 to 3.9 after application of 112 kg N fertilizer per hectare for 10 years in nearby region of Jiangxi province. After 26 years of intensive management, the decrease of pH values in A horizon ranges from 0.4 to 1.1 units, while that of B horizon soil ranges from 0.2 to 0.3 units (Table 2).

The strongly acidified soils induced by long-term fertilizer application also might be favorable for root rot disease development [22]. The lowered soil pH can impair the soil conditions grown with Chinese hickory trees, while this species is likely grown in the slightly acid or calcareous soils naturally [5].

Significant loss of nutrient elements is another important factor. According to Tables 2 and 3, we can see that at least 21.8% for total nitrogen (N), 7.6% for total phosphorus (P), and 13.6% for total potassium (K) in A horizon were lost from 1982 to 2008, respectively. A lot of nutrient elements are carried away in the picking process of *C. cathayensis* fruits. Prior to the fruit harvest, the grasses under the trees in the orchards were all removed either by hands or through herbicides, and the fruits in the tree were knocked down with a pole by hand at harvest. It is reported that about 30.7, 5.1, and 37 kg ha of N, P, and K were removed annually when yearly production reached 1000 kg ha of *C. cathayensis* fruits [23]. Moreover, as understory shrubs and weeds are removed, the runoff erosion intensity increases consequently, which also speeds up the loss of nutrient elements and facilitates soil degeneration. As a result, the soils were strongly disturbed and faced great risk of soil erosion in the sloping land and especially occurred after leaf drop in autumn and weather conditions.

In addition, it has been reported that about 83% of degraded land on the earth is triggered by soil erosion [24]. In our study, high slope gradient of the *C. cathayensis* forest land (>25°, Table 1), quite long rainy season, the sandy soil texture, and extensive mode of fertilizing operation can lead to about 16.1 kg ha N loss and 4.2 kg ha P loss.

Therefore, the long-term intensive management can cause soil acidification, reduction of the alkyl C and O-alkyl C, and great loss of nutrient elements of soils under *C. cathayensis* forest. All of the above will result in degradation of soil fertility quality. Li et al. [2] found that the application of organic manures and covering of organic mulch in bamboo forest in neighboring areas raised the organic carbon content about 31.8% in forest soils. To slow down forest soil degradation, it is recommended to increase the use of organic manure and other organic amendments. The increased soil acidity should be ameliorated through lime application.

## 5. Conclusions

Our study showed that the long-term intensive management in the Chinese hickory orchards had caused severe degradation of the soils, such as increased soil acidity, and reduced SOC content and nutrient concentrations. Frequent application of chemical fertilizers would have contributed to the soil acidification. Clearance of understory shrubs and weeds and lack of manure application in the intensively managed *C. cathayensis* have resulted in a significant reduction of SOC. ^13^C NMR analysis indicated that the long term management may have resulted in the reduction of alkyl C content in the SOC. The soil concentrations of the total N, P, and K, particularly in the A horizon, showed a decreasing trend downwards with time. Therefore, there is an urgent need to optimize soil management of the Chinese hickory orchards to improve soil conditions. To sustain the soil fertility and productivity of soils under *C. cathayensis* forest, it is advocated that more organic manure should be used together with chemical fertilizers.

## Figures and Tables

**Table 1:** Basic information of three fixed sample plots.

Code	Village^a^	Latitude	Longitude	Aspect	Elevation (m)	Gradient	Texture	Structure
XQ	Xiaxu	N30°14′	E118°56′	East	675	30	Clay	Lumpy
DS	Zhichuan	N30°16′	E118°54′	South	555	35	Clay	Nutty
HL	Deng	N30°20′	E119°18′	North	287	30	Fine Clay	Granular

^a^Xiaxu, Zhichuan, and Deng villages belong to Xiaxu, Zhichuan, and Henglu townships, respectively.

**Table 2:** Changes in soil properties under *C. cathayensis* forest stands after 26 years  intensive management.

Soil layer	A horizon		B horizon	
Sampling year	1982	2008	1982	2008
pH	7.1 ± 0.13^a^	6.4 ± 0.26^b^	6.9 ± 0.20^a^	6.4 ± 0.15^a^
SOC (g kg^−1^)	25.03 ± 0.84^a^	20.12 ± 0.13^b^	17.56 ± 1.72^a^	14.92 ± 0.67^a^
Total N (g kg^−1^)	2.52 ± 0.09^a^	1.97 ± 0.11^b^	1.86 ± 0.43^a^	1.56 ± 0.37^a^
Total P (g kg^−1^)	0.66 ± 0.36^a^	0.61 ± 0.34^a^	0.52 ± 0.28^a^	0.48 ± 0.28^a^
Total K (g kg^−1^)	6.09 ± 1.45^a^	5.26 ± 1.23^a^	5.99 ± 1.28^a^	5.18 ± 1.28^a^

Notes: for each soil horizon, values within a row followed by the same letter do not differ significantly.

**Table 3:** Property statistics of the soils under in *C. cathayensis* forest stands sampled during a soil survey in 1984 (unpublished data, Lin'an Agricultural Bureau).

Soil properties	Grade 1	Grade 2	Grade 3	Grade 4
pH	<6.0	6.0–6.5	6.5–7.0	>7.0
%	5.6	50.0	33.3	11.1
SOC (g kg^−1^)	<15.0	15.0–20.0	20.0–25.0	>25.0
%	38.9	38.9	11.1	11.1
Total N (g kg^−1^)	<1.0	1.0–1.5	1.5–2.0	>2.0
%	16.7	11.1	27.8	44.4
Total P (g kg^−1^)	<0.5	0.5–1.0	1.0–1.5	>1.5
%	66.7	11.1	16.7	5.6
Total K (g kg^−1^)	<3.0	3.0–5.0	5.0–7.0	>7.0
%	5.6	33.3	55.6	5.6

**Table 4:** Distributions of different chemical shift ranges in total signal intensity (%) for ^13^C NMR in organic carbon of A horizon soil under a *C. cathayensis* forest stand sampled in 1982 and 2008.

Year	Alkyl C (%)	O-alkyl C (%)	Aromatic C (%)	Carbonyl C (%)	A/O-A	Aromaticity (%)
1982	29.93^a^	46.41^a^	12.82^a^	10.83^b^	0.64	14.38^b^
2008	20.83^b^	49.19^a^	15.97^a^	14.00^a^	0.42	18.57^a^

Note: the dissimilar letters in the same column indicate significant difference at *P* < 0.05 level.
